# Cell cycle dependence of apoptosis photo-triggered using peptide-photosensitizer conjugate

**DOI:** 10.1038/s41598-020-76100-7

**Published:** 2020-11-05

**Authors:** Hyungjin Kim, Sho Watanabe, Mizuki Kitamatsu, Kazunori Watanabe, Takashi Ohtsuki

**Affiliations:** 1 Department of Interdisciplinary Science and Engineering in Health Systems, Okayama University, 3-1-1 Tsushimanaka, Okayama, 700-8530 Japan; 2 Department of Applied Chemistry, Kindai University, 3-4-1 Kowakae, Higashi-Osaka, Osaka 577-8502 Japan; 3 Present Address: Department of Organ Anatomy and Nanomedicine, Yamaguchi University, 1-1-1 Minami-Kogushi, Ube, 755-8505 Japan

**Keywords:** Biological techniques, Biotechnology

## Abstract

Investigation of the relevance between cell cycle status and the bioactivity of exogenously delivered biomacromolecules is hindered by their time-consuming cell internalization and the cytotoxicity of transfection methods. In this study, we addressed these problems by utilizing the photochemical internalization (PCI) method using a peptide/protein-photosensitizer conjugate, which enables immediate cytoplasmic internalization of the bioactive peptides/proteins in a light-dependent manner with low cytotoxicity. To identify the cell-cycle dependent apoptosis, a TatBim peptide-photosensitizer conjugate (TatBim-PS) with apoptotic activity was photo-dependently internalized into HeLa cells expressing a fluorescent ubiquitination-based cell cycle indicator (Fucci2). Upon irradiation, cytoplasmic TatBim-PS internalization exceeded 95% for all cells classified in the G_1_, S, and G_2_/M cell cycle phases with no significant differences between groups. TatBim-PS-mediated apoptosis was more efficiently triggered by photoirradiation in the G_1_/S transition than in the G_1_ and S/G_2_/M phases, suggesting high sensitivity of the former phase to Bim-induced apoptosis. Thus, the cell cycle dependence of Bim peptide-induced apoptosis was successfully investigated using Fucci2 indicator and the PCI method. Since PCI-mediated cytoplasmic internalization of peptides is rapid and does not span multiple cell cycle phases, the Fucci-PCI method constitutes a promising tool for analyzing the cell cycle dependence of peptides/protein functions.

## Introduction

The eukaryotic cell cycle is divided into gap 1 (G_1_), synthesis (S), gap 2 (G_2_), and mitotic (M) phases. The cell cycle dependence of various cell functions has long attracted considerable research attention. For example, the cell cycle dependence of antigen protein expression^1^, Ca^2+^ oscillation^2^, cell differentiation^3^, and cellular body formation^4^ has been evaluated. In addition, numerous researchers have studied the cell cycle dependence of cell fates induced by X-ray^5^, heat^6^, and drugs/chemicals^7,8^.

In contrast to cell permeable drugs/chemicals, investigation of the cell cycle dependence of cell fates induced by cell-impermeable peptides/proteins has been challenging because the most of peptide/protein transduction^9^ or peptide/protein gene transfection^10^ methodologies are time-consuming and the cell cycle is likely to progress to the next phase during these extended transduction or transfection procedures. In addition, the gene expression obtained following gene transfection is likely to continue throughout several cell cycle phases. In cultured mammalian cells, each cell cycle phase tends to last for tens of minutes to several hours^8,11^; for example, HeLa cells spend approximately 4.6 h in G_1_ phase, 6.0 h in S phase, 3.1 h in G_2_ phase, and 0.7 h in M phase; mouse embryonic stem cells spend approximately 1.0, 5.9, 2.7, and 0.6 h in the respective phases. Therefore, to investigate peptide/protein function during a specific cell cycle phase, a rapid peptide/protein transduction method is necessary. However, although electroporation and microinjection fulfill this requirement, the damage associated with the electrical stimulation or injection^12^ render it difficult to observe natural cellular responses immediately following the transduction.

**Figure 1:**
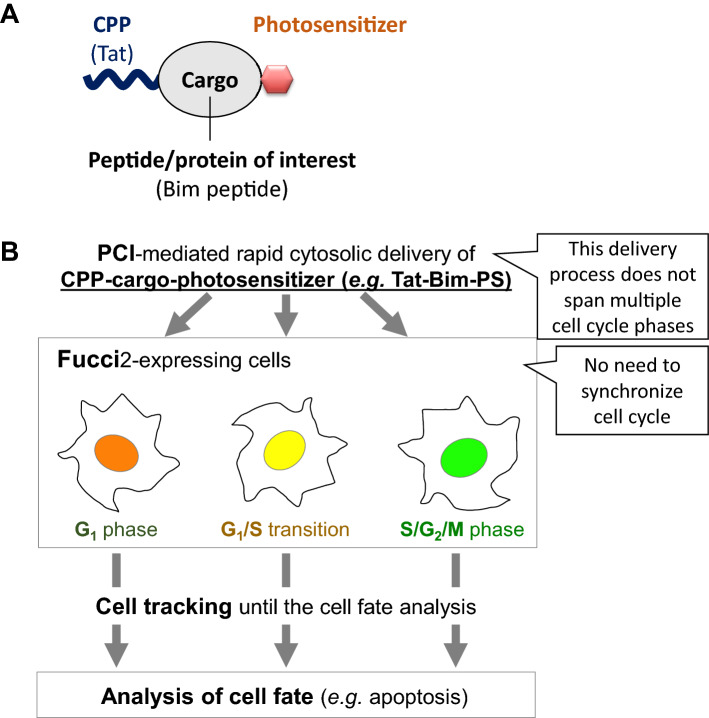
A CPP-cargo-photosensitizer conjugate and the Fucci-PCI method. (**A**) Design of a CPP-cargo-photosensitizer conjugate for PCI-mediated cytoplasmic peptide/protein delivery. The CPP, cargo, and photosensitizer used in this study are shown in parentheses. (**B**) A schematic overview of the Fucci-PCI method.

As an alternative, the photochemical internalization (PCI) method, which enables rapid cytoplasmic introduction (< 5 min after light irradiation) of a peptide/protein^13–19^, shows promise for investigating peptide/protein function in a specific cell cycle phase. PCI constitutes a method for inducing the light-directed endosomal escape (and cytoplasmic release) of macromolecules, which are delivered into cells through endocytosis and entrapped in endosomes^20,21^. In this method, endosomal membranes are destabilized by singlet oxygen photogenerated from photosensitizers^13,22,23^. For PCI-mediated peptide/protein delivery, cell penetrating peptide (CPP)-cargo-photosensitizer conjugates, in which cargo represents a peptide/protein of interest, can be used (Fig. 1A)^24–26^. Notably, through the use of photosensitizers with low singlet oxygen quantum yield combined with minimum light dose, photocytotoxicity can be very low^27^.

In this study, we investigated the relationship between the cell cycle and apoptosis induced by the Bim peptide delivered using the PCI method. Specifically, Bim peptide constitutes the BH3 domains of the Bim apoptosis-inducing protein, which serves as the biological effector domain^28,29^. For PCI, we used TatBim-PS^25,30^ (Fig. 1), a fusion molecule of the TAT cell-penetrating peptide^31,32^, the Bim peptide, and the photosensitizer Alexa Fluor 633 (Alexa633). The cell cycle was monitored using a fluorescent ubiquitination-based cell cycle indicator 2 (Fucci2) probe^33^. Here, we provide a new method for investigating the cell cycle dependence of peptide/protein function using Fucci2 imaging and rapid PCI-mediated cytoplasmic delivery of the peptide/protein (Fucci-PCI method) (Fig. 1B). This method enables investigation of the relationship between cell cycle phases and fate of the PCI-treated cells without necessitating a cell cycle-synchronization process. The novelty of this study lies in the combination of Fucci2-expressing cells that visualize some cell cycle states and the conjugated peptide that potentially shows a cell cycle-specific biological activity.

## Methods

### Preparation of the photosensitizer-conjugated TatBim (TatBim-PS)

TatBim peptide with a cysteine residue at the C-terminus (RKKRRQRRR-EIWIAQELRRIGDEFNAYYARLL-C) was prepared by solid-phase peptide synthesis using standard Fmoc protocols. Alexa Fluor 633 C5 maleimide (Life Technologies, Carlsbad, CA, USA), a photosensitizer, was then attached to the thiol group of the C-terminal Cys of the TatBim peptide. This reaction was performed at a peptide:Alexa633 molar ratio of 1:2 in dimethylformamide at 37 °C for 2 h. TatBim-PS (TatBim-Alexa633) was purified from the reaction mixture by reversed-phase high performance liquid chromatography (HPLC) on a C18 column using a linear 0–100% gradient of acetonitrile in 0.1% trifluoracetic acid (Fig. S1).

### Photoinduced cytoplasmic dispersion of TatBim-PS at specific cell-cycle phases

HeLa/Fucci2 cells^33^ were purchased from RIKEN BioResource Research Center (Tsukuba, Ibaraki, Japan). The cells were seeded at 2.0 × 10^4^ cells/well in 96 well plates and cultured overnight in RPMI 1640 medium supplemented with 10% fetal bovine serum (Sigma, St. Louis, MO, USA), 100 U/mL penicillin and 100 μg/mL streptomycin. The cells were then treated with 2 μM TatBim-PS for 2 h at 37 °C in serum-free RPMI 1640 medium. After washing twice with the serum-free medium, the cells in the medium were photoirradiated at 620 ± 25 nm at a fluence of 40 J/cm^2^ with a 100 W mercury lamp (U-LH100HG, Olympus, Tokyo, Japan)) passed through the Cy5 mirror unit and a 40 × objective lens from a fluorescence microscope (IX51, Olympus). Fluorescence images were acquired using a confocal laser scanning microscope (FLUOVIEW FV1000, Olympus). Fluorescence images were obtained through a 60 × objective lens with the following settings: Alexa633 (λ_ex_ = 633 nm, λ_em_ = 655–755 nm), mCherry–hCdt1 (λ_ex_ = 543 nm, λ_em_ = 555–625 nm), and mVenus–hGem (λ_ex_ = 488 nm, λ_em_ = 500–530 nm).

The cell cycle of each HeLa/Fucci2 cell was classified into one of three phases according to the Fucci2 color in the cell nucleus: orange/red for G_1_, green for S/G_2_/M, and yellow (merge of both of orange/red and green fluorescence) for G_1_/S (transition state from G_1_ to S). The cell cycle phase of each cell was classified using the cellular fluorescence images immediately following the photostimulation (time point 0 min after irradiation). The photoinduced cytoplasmic internalization efficiencies (%) of TatBim-PS at the corresponding cell-cycle phases were calculated by counting the number of cells in which Alexa633 fluorescence was dispersed within the cytosol after photostimulation (N_AF_) and the total cell number of the corresponding cell cycle (N_CC_). The cytoplasmic internalization efficiency at a given specific cell-cycle phase was defined as N_AF_/N_CC_ × 100 (%).

### Evaluation of the cell cycle-dependence of TatBim-PS-induced apoptosis

HeLa/Fucci2 cells were subjected to the same treatment as described above. Following irradiation, the cells were incubated in serum-free RPMI 1640 medium and time-lapse images were obtained every 30 min. The cell cycle phase of each cell was classified using the cellular fluorescence images obtained immediately following the photoirradiation. At 5 h after photoirradiation, the NucView405 Caspase-3 Substrate (Biotium, Fremont, CA, USA) was added to the medium at a final concentration of 100 μM to stain apoptotic cells. Blue fluorescence images were captured beginning at 30 min after the NucView405 addition using the FLUOVIEW FV1000 confocal laser scanning microscope.

Cell-cycle dependent apoptosis rates (%) were calculated by counting the number of cells with blue fluorescence (NucView405) at 5.5 h after photostimulation (N_BF_) and the N_CC_. Cell-cycle dependent apoptosis rate (%) was defined as N_BF_ / N_CC_ × 100 (%).

### Cell cycle synchronization with thymidine

HeLa/Fucci2 cells were treated with 2 mM thymidine in RPMI 1640 medium supplemented with 10% fetal bovine serum for 18 h for cell cycle synchronization. The medium including thymidine was removed and the cells washed twice with serum-free RPMI 1640 medium. The cells were then treated with TatBim-PS for 2 h and irradiated as described above.

### Statistical analysis

Student t-test was used for analyzing statistical differences between two groups. Comparison between more than two groups was performed by ANOVA followed by Fisher's test to determine significance of difference between all paired combinations. The *p* values less than 0.05 were considered to be statistically significant.

## Results and discussion

### Photoinduced cytoplasmic dispersion of TatBim-PS at each cell-cycle phase

TatBim-PS was prepared by attaching Alexa Fluor 633 C5 maleimide, a photosensitizer, to the C-terminal Cys of the TatBim peptide. HPLC-purified TatBim peptide carried Alexa633 photosensitizer to the extent of 50%; this was used as TatBim-PS in subsequent experiments.

**Figure 2:**
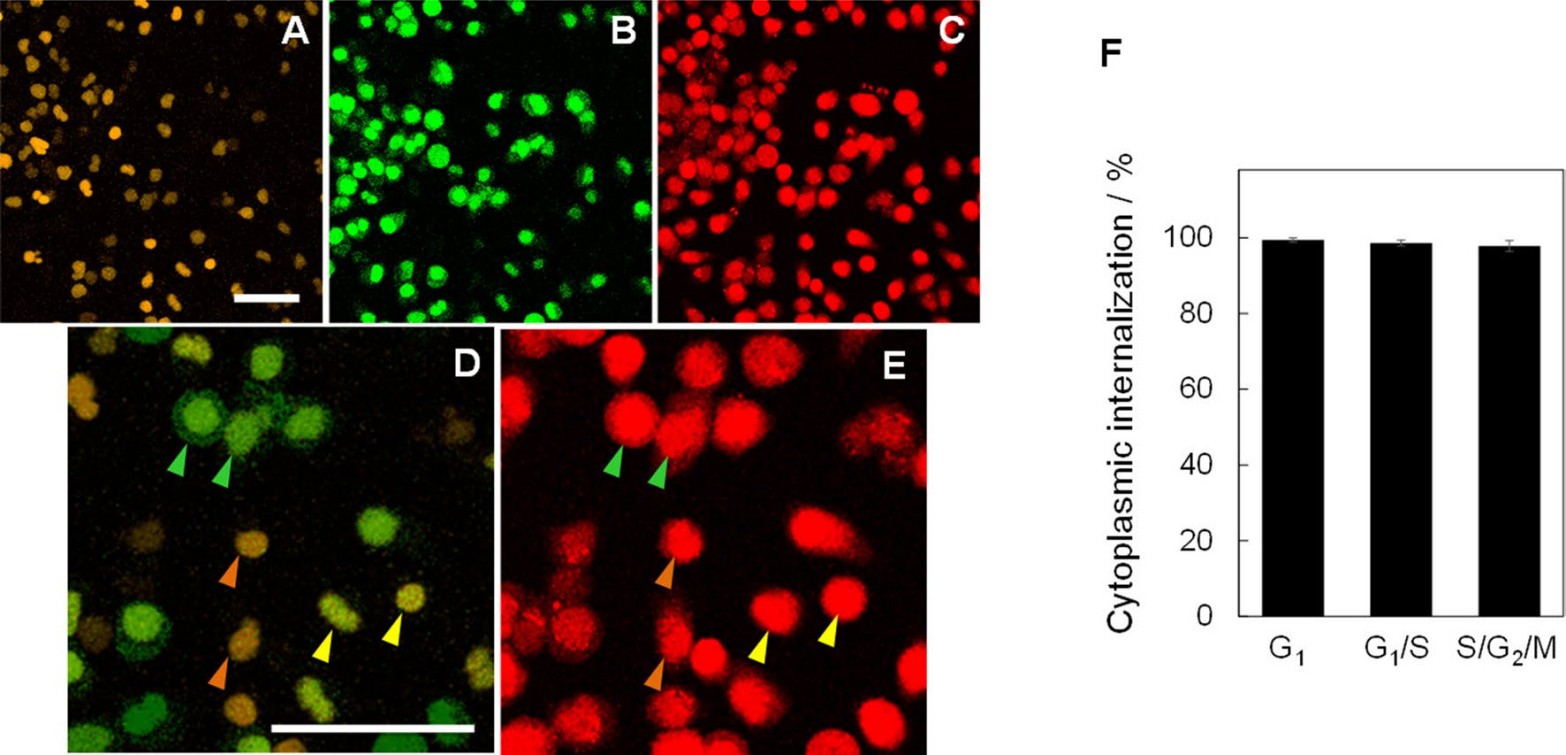
Efficient internalization of TatBim-PS by HeLa/Fucci2 cells irrespective of cell-cycle phase. **(A–C)** Fluorescence images of the mCherry–human Cdt1 fusion protein (mCherry–hCdt1) showing G_1_ and G_1_/S transition phases **(A)**, mVenus–human Geminine fusion protein (mVenus–hGem) showing S/G_2_/M phase **(B)**, and TatBim-PS **(C)**. The fluorescence of mCherry (emission maximum around 610 nm) was pseudo-colored as orange to distinguish it from the red fluorescence of TatBim-PS (PS = Alexa633; emission maximum at around 650 nm). Scale bar, 50 µm. **(D)** Magnified, merged image of A and B. Green arrows: S/G_2_/M, Orange arrows: G_1_, and, Yellow arrows: G_1_/S transition. Scale bar, 50 µm. **(E)** Magnified image of C. The arrows indicate the same cell cycle phases as in D, with identical color scheme. **(F)** Cytoplasmic internalization efficiency at each cell-cycle. Data are shown as the mean ± SEM (n = 3; each of the analyzed areas included 117 cells on average).

HeLa/Fucci2 cells were treated with TatBim-PS for 2 h. This procedure induces endocytosis and endosomal entrapment of TatBim-PS^25^. After the TatBim-PS treatment, the cells were irradiated at the excitation wavelength (620 ± 25 nm) of the photosensitizer Alexa633 for inducing endosomal escape and cytoplasmic dispersion of TatBim-PS by the PCI mechanism^13,25,26^. We first evaluated the light dose-dependence of TatBim-PS cytoplasmic dispersion (Fig. S2); the light dose for TatBim-PS internalization by HeLa/Fucci2 cells was determined as 40 J/cm^2^. Immediately after the irradiation, the cells were imaged by confocal laser scanning fluorescence microscopy (Fig. 2A–D). The cell cycle phase of each cell was determined using Fucci2 imaging. Specifically, the cells displaying mCherry–hCdt1 fluorescence were classified as G_1_ (Fig. 2A), with the green fluorescence of mVenus–hGem were classified as S/G_2_/M (Fig. 2B), and cells displaying both orange and green fluorescence (yellow in the merged image (Fig. 2D)) were classified as G1/S; that is, in the transition state from G1 to S. Upon irradiation, cytoplasmic dispersion of Alexa633 fluorescence derived from TatBim-PS was confirmed in the majority of cells (Fig. 2C,E). We then analyzed cell cycle-dependence of the cytoplasmic internalization efficiency of TatBim-PS. Cytoplasmic internalization efficiency was over 95% for all cells in the three different cell cycle phases and did not markedly differ between the groups (Fig. 2F).

### Cell cycle dependence of apoptosis induced by TatBim-PS

**Figure 3:**
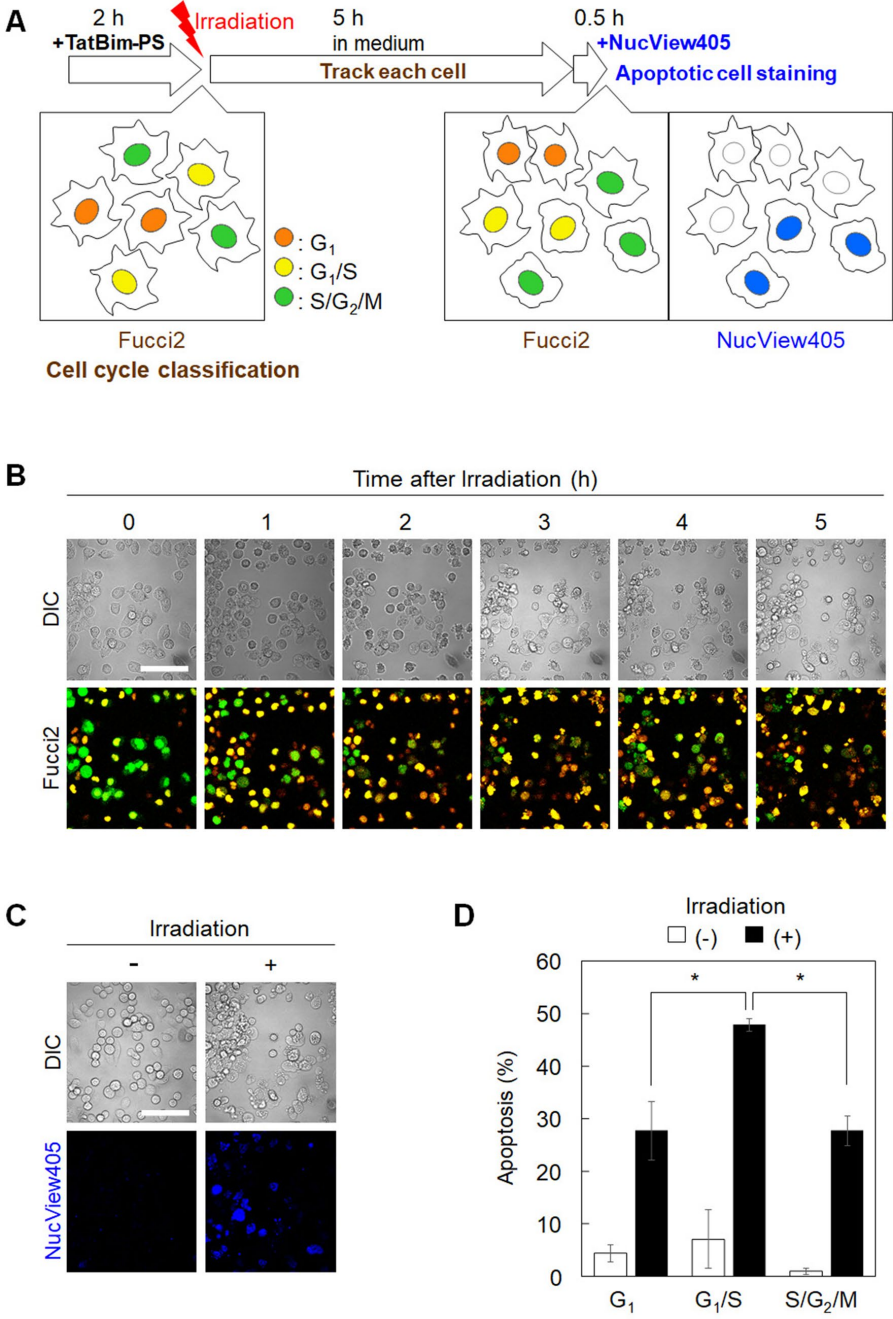
TatBim-PS induces apoptosis by photoirradiation most efficiently in the G_1_/S transition phase compared to that in other cell-cycle phases. **(A)** Scheme of the Fucci-PCI method to investigate cell-cycle-dependent apoptosis. First, HeLa/Fucci2 cells were treated with TatBim-PS for 2 h, and thereafter photoirradiated. Following the irradiation, the cells were incubated and time-lapse images were obtained using the same field of view. The cell cycle phase of each cell was classified using the cellular fluorescence images obtained immediately following the irradiation (left panel). Five hours after photoirradiation, apoptotic cells were stained with NucView405 for 30 min. After the staining, blue NucView405 image (right panel) was captured at the same field as the left and middle panels. **(B)** Cell-cycle tracking every hour following photoirradiation (TatBim-PS internalization). **(C)** Light-dependent apoptosis of HeLa/Fucci2 cells at 5.5 h following photoirradiation. Scale bar, 50 µm. **(D)** Apoptosis rate of cells at each cell cycle phase. Data are shown as the mean ± SEM (n = 3; each of the analyzed areas included 111 cells on average). The *p* value was determined by one-way ANOVA.

To investigate the cell-cycle dependence of TatBim-mediated apoptosis, HeLa/Fucci2 cells were tracked for 5.5 h following photoinduced cytosolic dispersion of TatBim-PS (Fig. 3A,B). The cell cycle phase of each cell was classified at the time point of 0 h. Cell tracking was performed as shown in Figure S3. At 5 h after irradiation, apoptotic cells were stained for 30 min with NucView 405 Caspase-3 Substrate, which emits fluorescence in response to caspase-3/7 activity. Apoptotic cells were observed to occur in a light- (Fig. 3C) and photosensitizer- (Fig. S4) dependent manner, indicating that both light and the photosensitizer are necessary for TatBim-induced apoptosis. CCK-8, LDH, and JC-1 assays supported that cell death (or decrease of cell viability) could be induced by TatBim-PS and light treatment, but not by photosensitizer Alexa633 and light treatment (data not shown). The phototoxicity was slightly observed (Fig. S4). However, this slight phototoxicity did not seem to significantly affect the cell cycle phase selectivity of TatBim-PS activity as the apoptosis ratio of irradiated cells differed less than 2% among the cell-cycle phases (Fig. S4B). TatBim-PS induced apoptosis by photoirradiation in the G_1_/S transition phase (47%) more efficiently than in the G_1_ (27%) and S/G_2_/M (27%) phase (Fig. 3D). This result indicated that the cells in the G_1_/S transition phase exhibit high sensitivity to Bim-induced apoptosis. In contrast, no significant correlation was observed between TatBim-PS-induced apoptosis and cell cycle phase at 5.5 h following irradiation (Fig. S5), implying that the cell cycle phase is important for apoptosis induction only at the time point of cytoplasmic introduction of TatBim-PS but not subsequent time points.

### Cell cycle synchronization with thymidine

**Figure 4:**
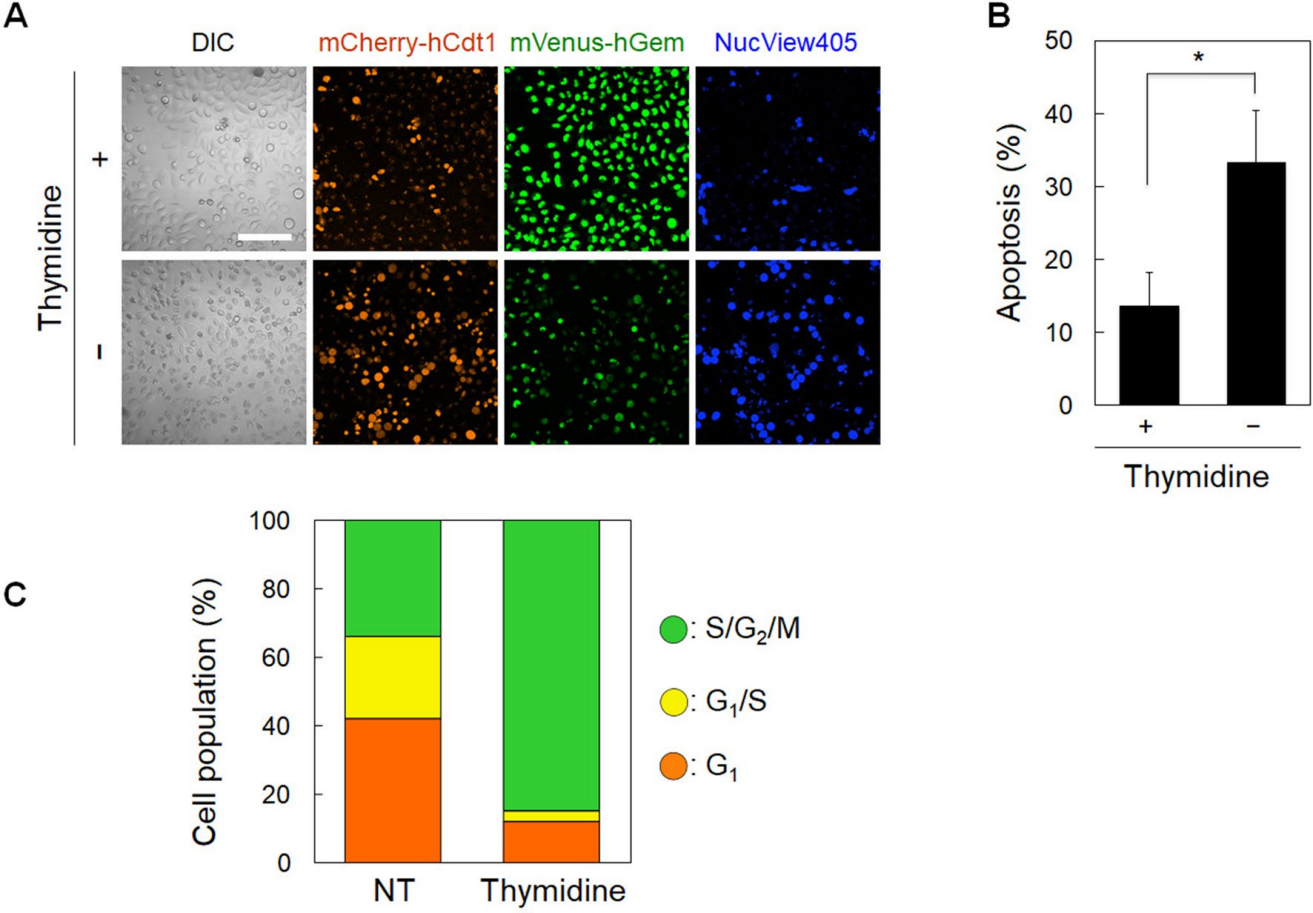
Decrease of cell population of the G_1_/S transition phase reduced the apoptosis rate. TatBim-PS induced apoptosis efficiently in the G_1_/S transition phase. **(A)** Cell cycle population and light-dependent apoptosis after treatment with thymidine. *Scale bar*, 50 µm. **(B)** Apoptosis rate after the treatment with thymidine. Data are shown as the mean ± SEM (n = 3; each of the analyzed areas included 123 cells on average). *p < 0.05 by analysis of variance followed by Student's t-test. **(C)** Cell cycle population after thymidine treatment. Data are shown as the mean (n = 3; each of the analyzed areas included 118 cells on average).

To confirm that TatBim-PS efficiently induced apoptosis in the G_1_/S transition phase, we decreased the rate of G_1_/S transition phase cell formation using thymidine and examined the TatBim-PS-induced apoptosis rate of the HeLa/Fucci2 cells. Thymidine is a DNA synthesis inhibitor that can arrest cells at the G_1_/S boundary and, thus, it can synchronize cells at the S phase after its removal. The cells treated with thymidine followed by 2 h TatBim-PS treatment were mainly detected in the S/G_2_/M phase rather than the G_1_/S transition phase (Fig. 4A). This result indicated that the thymidine-treated cells were mostly synchronized to the S phase, as described previously^34,35^. Specifically, Velichko et al.^35^ reported that HeLa cells are synchronized to the early S phase at 2 h following thymidine treatment, which is similar to the timing of our cell cycle observations (approximately 2.2 h following thymidine treatment). The apoptosis rate among the total cells was significantly decreased from 33% (non-treated cells) to 14% (thymidine-treated cells) (Fig. 4B). As the rate of the G_1/_S transition phase decreased to only 3% of the total cell number by the thymidine treatment (Fig. 4A), the observed reduced apoptosis rate in the thymidine-treated cells (Fig. 4B) supports the concept that TatBim-PS induces apoptosis more efficiently in the G_1_/S transition phase than in the other phases.

### Bim and the G_1_/S transition phase

The reason why TatBim-PS-mediated apoptosis was enhanced in the G_1_/S transition phase remains unclear. Several reports indicate a relationship between Bim and the G_1_/S checkpoint. For example, deregulation of Cdk2, which is a key regulator of the G_1_/S checkpoint^36,37^, causes Foxo1 activation followed by Bim-mediated apoptosis^38,39^. Such pathways might be associated with the enhanced Bim-mediated apoptosis in the G_1_/S transition phase. However, our experiments were initiated upon cellular internalization of Bim (TatBim-PS), which is located downstream of the G_1_/S checkpoint-related factors in the apoptosis pathway. Thus, it is difficult to consider that exogenous Bim affects an upstream factor (Cdk2 or Foxo1) which subsequently enhances Bim-mediated apoptosis. There might be some degree of feedback involved in the pathway.

Alternatively, Wan et al.^40^ reported that the Bim expression level increases during the G_1_ and early S phases compared to that in the late S and G_2_/M phases. Although Bim upregulation is not G_1_/S transition phase-specific but also occurs in the G_1_ phase, a difference of endogenous Bim concentration in each cell cycle phase might be related to the enhancement of TatBim-PS-mediated apoptosis in the G_1_/S phase. Thus, additional experiments such as the induction of Bim-mediated apoptosis in the presence of inhibitors of G_1_/S checkpoint-related factors or factors upregulated at the G_1_/S transition phases will be necessary to address the mechanisms underlying the observed effects.

### Consideration of the methods for studying the cell cycle-dependence of peptide/protein functions

In this study, we analyzed the cell cycle-dependence of the apoptotic function of Bim peptide via the Fucci-PCI method based on Fucci2 imaging of PCI-treated (Bim peptide-introduced) cells followed by single cell tracking (Fig. 3). The target peptide (Bim) was conjugated with the Tat cell-penetrating peptide and a photosensitizer for PCI-mediated quick delivery into the cytosol.

Compared to the method based on cell cycle synchronization (Fig. 4), the Fucci-PCI method presents several advantages, including: (1) the peptide function in every cell cycle phase can be analyzed under a single experimental condition; and (2) the undesired effects of cell cycle synchronizing reagents can be avoided. In contrast, a disadvantage of the Fucci-PCI method is that some phases (in the case of Fucci2, S, G_2_, and M phases) cannot be distinguished. In this study, the TatBim-PS/photo-induced apoptosis rate determined from the Fucci-PCI method was 27% in the majority of cell cycle phases except for in the G_1_/S transition phase (Fig. 3D), whereas the thymidine treatment reduced the apoptosis rate to a greater degree than expected (14%) (Fig. 4B), possibly due to the undesired effects of thymidine other than the S phase synchronization effect.

## Conclusion

Cell cycle dependence of Bim peptide-induced apoptosis was successfully investigated using TatBim-PS and HeLa/Fucci2 cells. Upon photoirradiation, TatBim-PS was efficiently internalized by HeLa/Fucci2 irrespective of cell cycle stage. Apoptosis was most efficiently induced at the G_1_/S transition phase compared to that of G_1_ and S/G_2_/M phases. S-phase synchronization by thymidine treatment decreased the cell population of the G_1_/S state and resulted in reduction of the apoptosis rate, demonstrating that the cells in the G_1_/S state are highly sensitive to Bim-mediated apoptosis. The Fucci-PCI method thus appears promising for analyzing the cell cycle dependence of functions of peptides/proteins (other than Bim). Although this study used HeLa/Fucci2 cells only, the Fucci-PCI method can be applied to numerous cell lines transformed by the commercially available Fucci gene. It is particularly easy to use already available Fucci-expressing cell lines, such as NMuMG/Fucci and COS/Fucci in the RIKEN cell bank. Moreover, the system may also potentially be applied for studying the cell cycle dependence of RNA functions as a photoinduced cytoplasmic RNA delivery system has been developed^27,41,42^.

## Supplementary information

Supplementary Information.
