# Supplementary material for: Cell cycle dependence of apoptosis photo-triggered using peptide-photosensitizer conjugate
Source: Sci Rep. 2020 Nov 5;10:19087. doi: 10.1038/s41598-020-76100-7 (PMC7644668; doi:10.1038/s41598-020-76100-7)
Supplement: Supplementary file 1 — Supplementary Information. [file 41598_2020_76100_MOESM1_ESM.pdf]

Supporting Information to:

Cell cycle dependence of apoptosis photo-triggered using peptide-photosensitizer conjugate

Hyungjin Kim,[1,#] Sho Watanabe,[1] Mizuki Kitamatsu,[2] Kazunori Watanabe[1] and Takashi Ohtsuki[1,*]

[1] Department of Interdisciplinary Science and Engineering in Health Systems, Okayama University, 3-1-1 Tsushimanaka, Okayama 700-8530, Japan
[2] Department of Applied Chemistry, Kindai University, 3-4-1 Kowakae, Higashi-Osaka, Osaka 577-8502, Japan

[#] Present Address:
Department of Organ Anatomy and Nanomedicine, Yamaguchi University, 1-1-1 Minami-Kogushi, Ube 755-8505, Japan

* To whom should be correspondence
E-mail: ohtsuk@okayama-u.ac.jp

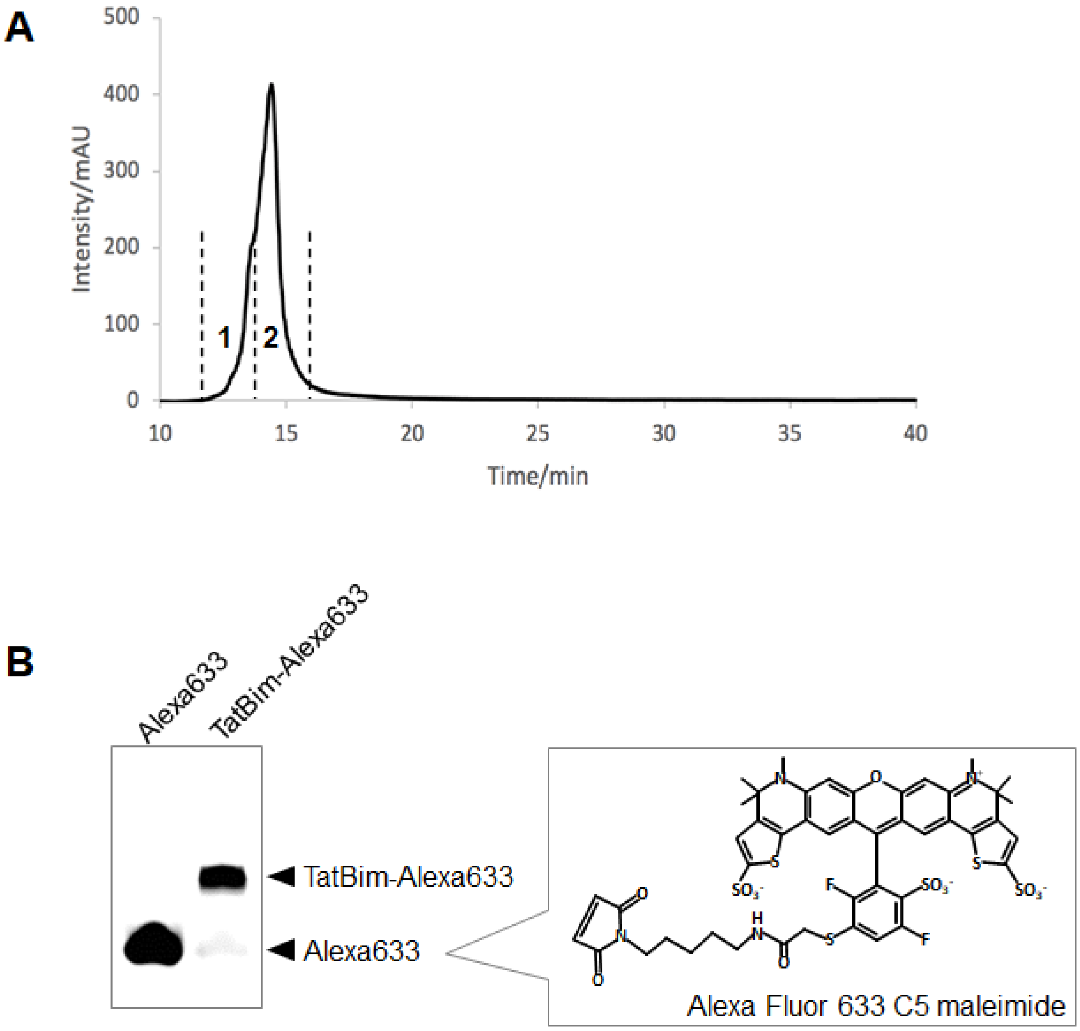

Figure S1. Preparation of TatBim-Alexa633 (TatBim-PS). (**A**) HPLC chart of the reacted solution for the TatBim-PS. The purification was performed by a reversed-phase HPLC (Symphonia C18 Column [4.6 × 150 mm, 5 μm particle diameter; Jasco, Tokyo, Japan]) eluted at a flow rate of 0.6 mL/min with a 40 min linear gradient of 0–100% actonitrile/H_2O in 0.1% trifluoracetic acid. The 2nd fraction (around the retention time of 15 min) was collected and used as TatBim-Alexa633. (**B**) 18% SDS-PAGE analysis of TatBim-Alexa633. The right panel shows the chemical structure of Alexa Fluor 633 C5 maleimide used in this study.

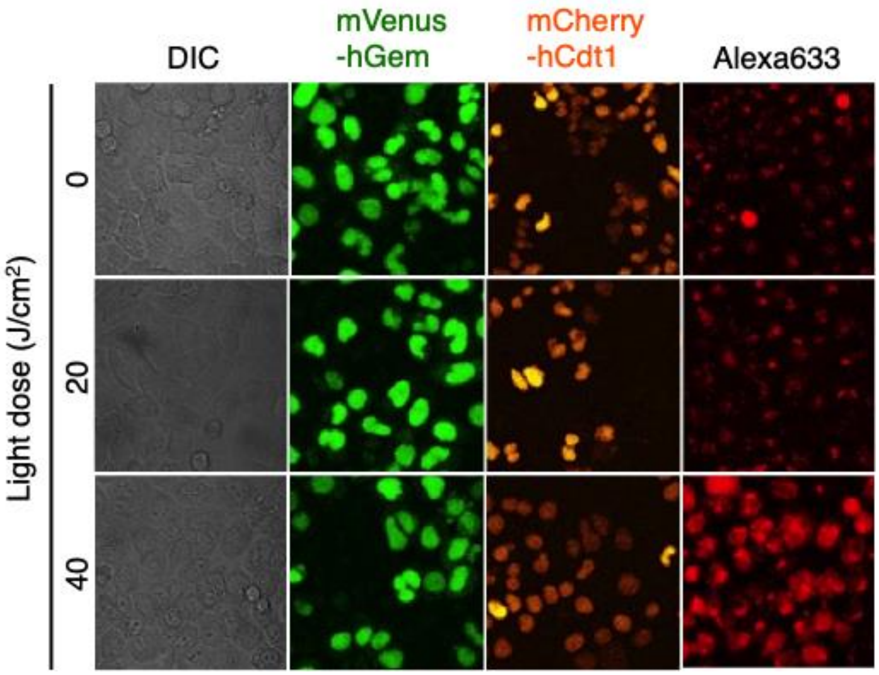

Figure S2. Light dose-dependency of cytoplasmic dispersion of TatBim-PS in HeLa/Fucci2 cells.

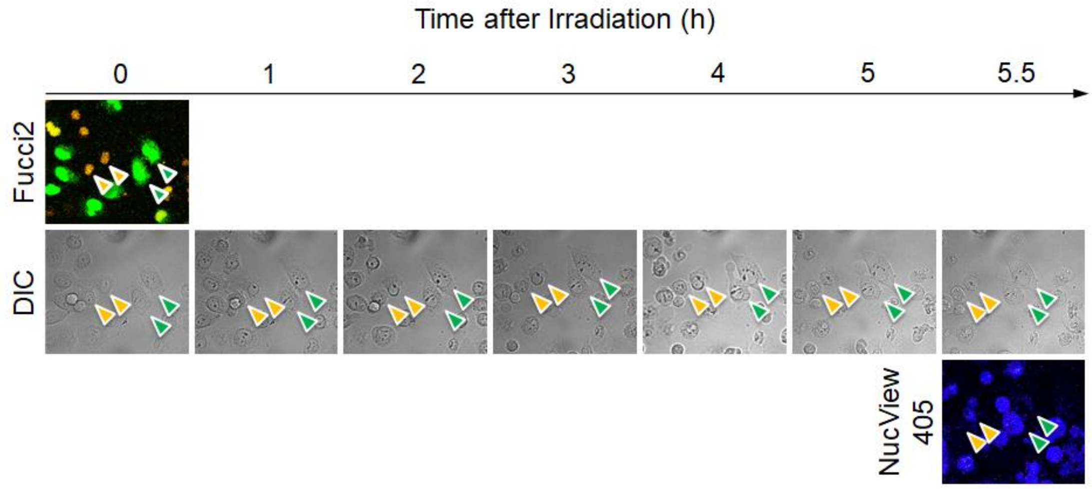

Figure S3. Cell tracking after irradiation. HeLa/Fucci2 cells treated with TatBim-PS were irradiated at 620 ± 25 nm at a fluence of 40 J/cm^2, and imaged by microscopy. Time-lapse images were obtained from the same area. At 5 h after photoirradiation, apoptotic cells were stained with NucView405 for 30 min. For example, two cells at G_1 phase and two cells at S/G_2/M phase (at the time zero point) are tracked by orange and green arrowheads, respectively. These images show an example of cell tracking for measuring apoptosis rate of cells at each cell cycle phase (Fig. 3D). Compared to these images, the images used for measuring Figure 3D data covered larger areas (n = 3; each of the analyzed areas included 111 cells on average).

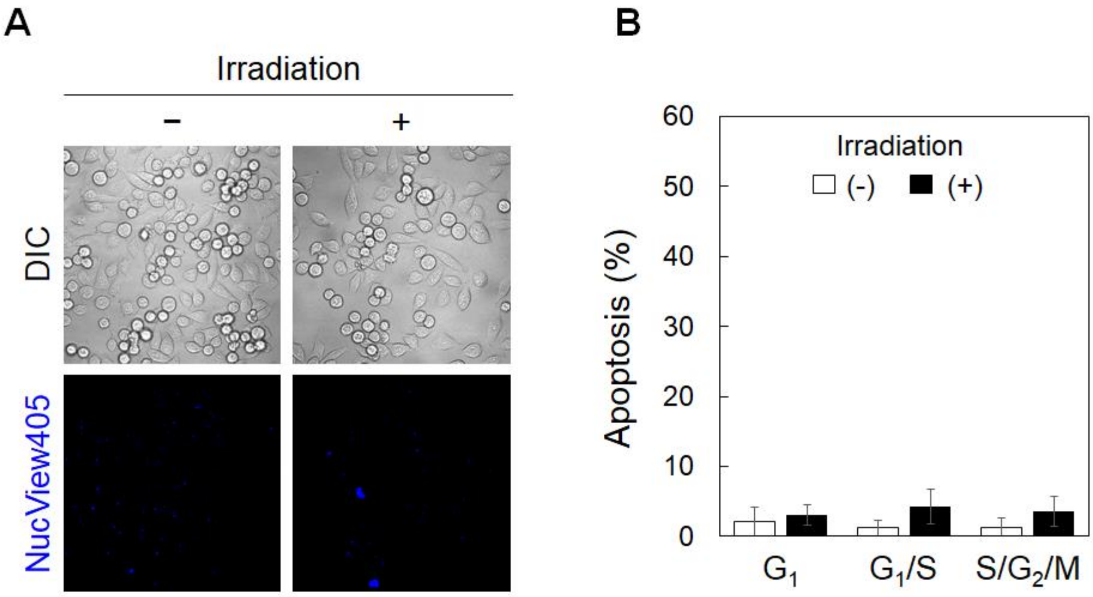

Figure S4. TatBim peptide without photosensitizer do not induce apoptosis even after light irradiation. (A) DIC and NucView405 (apoptosis) images of HeLa/Fucci2 cells treated with TatBim. **(B)** Apoptosis rate of cells at each cell cycle phase (at time point of 0 h).

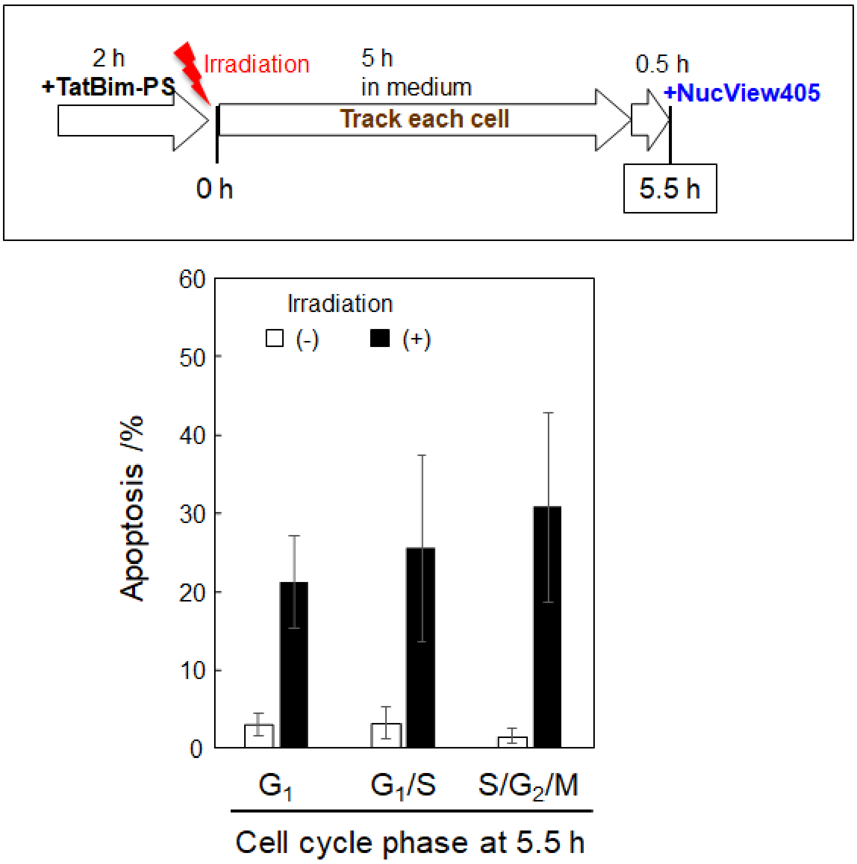

Figure S5. No correlation was observed between TatBim-PS-induced apoptosis and cell cycle phases at 5.5 h after irradiation. Data are shown as the mean ± SEM (n = 3). Five hours after irradiation, apoptotic cells were stained for 30 min with NucView 405 Caspase-3 Substrate.
